# Draft genome sequence of *Lactobacillus plantarum* strains E2C2 and E2C5 isolated from human stool culture

**DOI:** 10.1186/s40793-017-0222-x

**Published:** 2017-01-30

**Authors:** Mangesh V. Suryavanshi, Dhiraj Paul, Swapnil P. Doijad, Shrikant S. Bhute, Tejashri B. Hingamire, Rahul P. Gune, Yogesh S. Shouche

**Affiliations:** 1 Microbial Culture Collection, National Centre for Cell Science, Ganeshkhind, Pune, 411007 India; 2 Institute of Medical Microbiology, Biomedizinisches Forschungszentrum Seltersberg, Schubertstr. 81, Giessen, 35392 Germany; 3 Department of Zoology, Savitribai Phule Pune University, Ganeshkhind, Pune, 411007 India; 4 Biochemical Science Division, CSIR-National Chemical Laboratory, Homi Bhabha Road, Pune, 411008 India; 5 Department of Urology, RCSM Govt. Medical College, CPR Hospital Compound, Bhausingji Rd, Kolhapur, 416002 India

**Keywords:** Human Stool, Bacteria, Firmicutes, *Lactobacillus plantarum*

## Abstract

Probiotic *Lactobacillus* species offer various health benefits, thus have been employed in treatment and prevention of various diseases. Due to the differences in the isolation source and the site of action, most of the lactobacilli tested in-vitro for probiotics properties fail to extend similar effects in-vivo. Consequently, the search of autochthonous, efficacious and probably population specific probiotics is a high priority in the probiotics research. In this regards, whole genome sequencing of as many *Lactobacillus* as possible will help to deepen our understanding of biology and their health effects. Here, we provide the genomic insights of two coherent oxalic acid tolerant *Lactobacillus* species (E2C2 and E2C5) isolated from two different healthy human gut flora. These two isolates were found to have higher tolerance towards oxalic acid (300 mM sodium oxalate). The draft genome of strain E2C2 consists of 3,603,563 bp with 3289 protein-coding genes, 94 RNA genes, and 43.99% GC content, while E2C5 contained 3,615,168 bp, 3293 coding genes (93.4% of the total genes), 95 RNA genes and 43.97% GC content. Based on 16S rRNA gene sequence analysis followed by *in silico* DNA-DNA hybridization studies, both the strains were identified as *Lactobacillus plantarum* belonging to family *Lactobacillaceae* within the phylum *Firmicutes*. Both the strains were genomically identical, sharing 99.99% CDS that showed 112 SNPs. Both the strains also exhibited deconjugation activity for the bile salts while genome analysis revealed that the *L. plantarum* strains E2C2 and E2C5 also have the ability to produce vitamins, biotin, alpha- and beta- glucosidase suggesting potential probiotic activities of the isolates. The description presented here is based on the draft genomes of strains E2C2 and E2C5 which are submitted to GenBank under the accession numbers LSST00000000.1 and LTCD00000000.1, respectively.

## Introduction

The genome of lactobacilli is highly diversified which endorses them to occupy wide range of ecological habitats, including carbohydrate-rich environments [1], fermented meats [2], sourdoughs [3], plant-derived substrates [4] and different niches on and in the human body namely respiratory, gastrointestinal and urogenital tract [5, 6]. Owing to the beneficial effects offered by lactobacilli, they have been used as a gold standard in probiotic preparations. Consequently, many strains of lactobacilli such as *Lactobacillus acidophilus*, *L. amylovorus*, *L. brevis*, *L. bulgaricus*, *L. casei*, *L. fermentum*, *L. lactis*, *L. pentosus* *,* and *L. rhamnosus* have been well characterized for their ability to produce extracellular proteins, exopolysaccharides, and lipoteichoic acids, which influence the health and physiology of the host by interacting with the epithelial cells and enhancing the host immune system [7–12].

From the array of various *Lactobacillus* species, *Lactobacillus plantarum*, an organism found in a variety of ecological environments, is a well characterized probiotic species. Recent genome analysis of *Lactobacillus plantarum* WCFS1 indicates that this organism is endowed with sets of genes essential for survival in gastrointestinal tract, interactions with other organisms in the gut, interactions with the host epithelial barrier and immune system, making it an extremely versatile probiotic bacterium [13] and that the genome of this organism is highly plastic [14]. Despite the extraordinary features possessed by *L. plantarum*, it suffers from some drawbacks. First, a study involving the pharmacokinetics of *L. plantarum* has indicated that it is a transient passenger in the gut [15]. Secondly, significant genome editing is required in order to gain the improved probiotic properties [16]. Both of these could be attributed to the incompatibility of the isolation source e.g. human saliva [17] and its implied target (gut). Thus, the search of indigenous *L. plantarum* strains (e.g. from human gut) is a thrust area in probiotic research and its implications to human health.

Microbial communities in the human gut are complex and astonishingly diverse in nature [18]. Despite the fact that lactobacilli contribute minutely to these trillions of cells, due to their beneficial roles in gut ecology, they are gaining attention in biomedical research [19]. Consequently, we focused on the isolation of oxalate tolerant Lactobacilli from healthy stool samples. Out of the 16 *Lactobacillus* isolates grown on MRS media, two isolates E2C2 and E2C5 showed comparatively higher tolerance to oxalic acid and bile salt. Owing to the fact that hyperoxaluria leads to dysbiosis in the human gut [20], these strains of *L. plantarum*, GRAS category organism, may specifically be useful in ameliorating the hyperoxaluria and associated complications. We, therefore, sequenced the genomes of these isolates using Illumina Miseq platform and compared their metabolic potentials.

## Organism information

### Classification and features

The two oxalic acid tolerant isolates, E2C2 and E2C5, were isolated from human stool samples by double enrichment method (100 and 200 mM/L sodium oxalate) using MRS (10 g enzymatic digest of animal tissue, 10 g beef extract, 5 g yeast extract, 20 g dextrose, 5 g sodium acetate, 1 g polysorbate 80, 2 g potassium phosphate, 2 g ammonium citrate, 0.1 g magnesium sulfate, 0.05 g manganese sulfate) medium. These bacterial isolates were maintained on MRS agar at the incubation temperature of 30 °C and at pH 6.8.

**Fig. 1:**
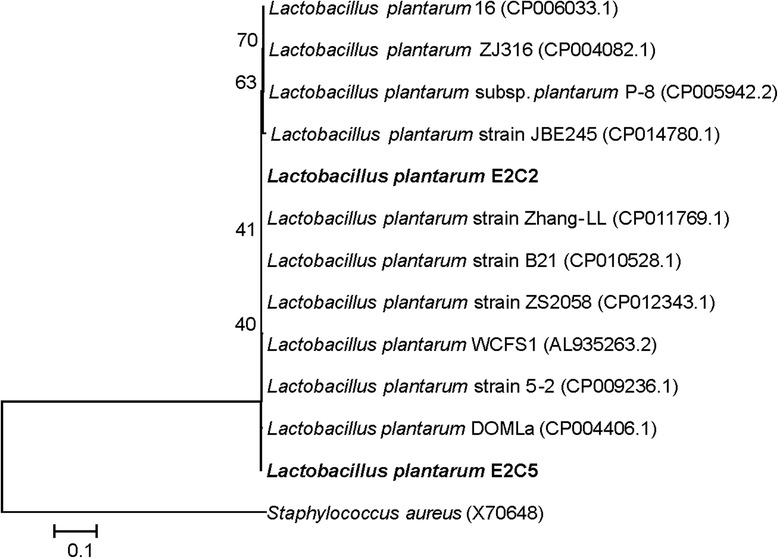
Neighbour-joining phylogenetic tree is constructed based on 16S rRNA gene sequence. The tree is constructed using Jukes–Cantor distances. Then 1000 bootstraps analyses are conducted. Sequences represented in bold font are derived from isolated strains of this study

**Fig. 2:**
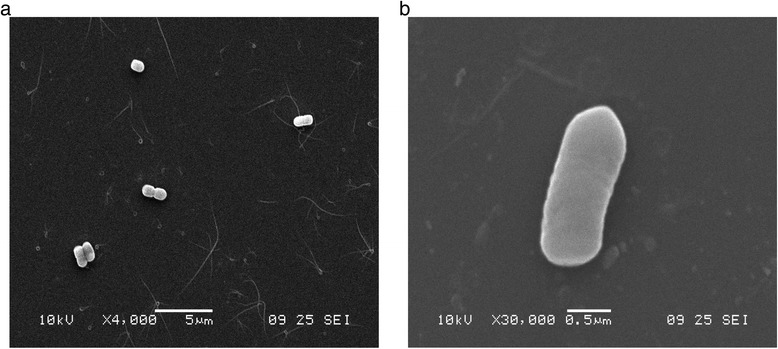
Scanning electron microscopic (SEM) analysis of bacterial isolates (**a**) *Lactobacillus plantarum* E2C2 and (**b**) *Lactobacillus plantarum* E2C5

The strains were tested for phenotypic and biochemical characterization (Table 1). *L. plantarum* E2C2 and E2C5 isolates are Gram-positive, non-motile, non-spore forming and rod-shape in morphology (Fig. 1 and Table 1). While, in the case of bile salts, both the strains could grow up to 0.40% w/v of Oxgall (Sigma-Aldrich) tested for 24 h incubation at 30 °C. It was observed that these isolates have the ability to deconjugate the glycodeoxycolate (bile salt) and this activity was confirmed by plate assay and TLC assay methods [21]. Ninhydrin assay [22] was performed to quantitate the bile salt hydrolase production ability which was found to be maximum at the 72 h, 5.22 U and 5.27 U for glycodeoxycholic acid as a substrate for E2C2 and E2C5 isolates, respectively (Fig. 2). They were able to utilize a large number of carbon compounds, namely dextrose, fructose, galactose, inulin, L-arabinose, maltose, mannose, mannitol, melibiose, Na-gluconate, raffinose, salicin, sorbitol, sucrose, trehalose, xylose, etc. during their growth (Table 1).

**Table 1:** Classification and general features of *L. plantarum* E2C2 and *L. plantarum* E2C5

MIGS ID	Property	*L. plantarum* E2C2	*L. plantarum* E2C5	Evidence code^a^
	Domain	Bacteria	Bacteria	TAS [41]
	Phylum	*Firmicutes*	*Firmicutes*	TAS [42, 43]
	Class	*Bacilli*	*Bacilli*	TAS [44]
	Order	*Lactobacillales*	*Lactobacillales*	TAS [45]
	Family	*Lactobacillaceae*	*Lactobacillaceae*	TAS [46]
	Genus	*Lactobacillus*	*Lactobacillus*	TAS [43, 47–50]
	Species	*Lactobacillus plantarum*	*Lactobacillus plantarum*	TAS [43, 47, 51]
	Strain	E2C2	E2C25	
	Gram stain	Positive	Positive	TAS [43]
	Cell shape	Rod	Rod	IDA
	Motility	non-motile	non-motile	TAS [43]
	Sporulation	spore forming	spore forming	IDA
	Temperature range	25 °C −39 °C	25 °C −39 °C	NAS
	Optimum temperature	30 °C	30 °C	TAS [43]
	pH range; Optimum	3.5–6.5; 5	3.5–6.5; 5	TAS [43]
	Carbon source	Xylose, Maltose, Fructose, Dextrose, Galactose, Raffinose, Melibiose, Trehalose, Sucrose, L-Arabinose, Mannose, Inulin, Na-gluconate, Salicin, Sorbitol, Mannitol, Cellobiose, Melezitose, ONPG, Esculin, Citrate, Malonate	Xylose, Maltose, Fructose, Dextrose, Galactose, Raffinose, Melibiose, Trehalose, Sucrose, L-Arabinose, Mannose, Inulin, Na-gluconate, Salicin, Sorbitol, Mannitol, Cellobiose, Melezitose, ONPG, Esculin, Citrate, Malonate	IDA
MIGS-6	Habitat	Human stool	Human stool	IDA
MIGS-6.3	Salinity tolerance	5- 8%	5- 8%	TAS [45]
MIGS-22	Oxygen requirement	Facultatively anaerobic	Facultatively anaerobic	TAS [43]
MIGS-15	Biotic relationship	Free-living	Free-living	TAS [45]
MIGS-14	Pathogenicity	non-pathogen	non-pathogen	NAS
MIGS-4	Geographic location	India/Asia	India/Asia	IDA
MIGS-5	Sample collection	November 2015	November 2015	IDA
MIGS-4.1	Latitude	18.5204° N	18.5204° N	IDA
MIGS-4.2	Longitude	73.8567° E	73.8567° E	IDA
MIGS-4.4	Altitude	562 m a.s.l.	562 m a.s.l.	IDA

^a^Evidence codes - IDA: Inferred from Direct Assay; TAS: Traceable Author Statement (i.e., a direct report exists in the literature); NAS: Non-traceable Author Statement (i.e., not directly observed for the living, isolated sample, but based on a generally accepted property for the species, or anecdotal evidence). These evidence codes are from the Gene Ontology project [52]

**Fig. 3:**
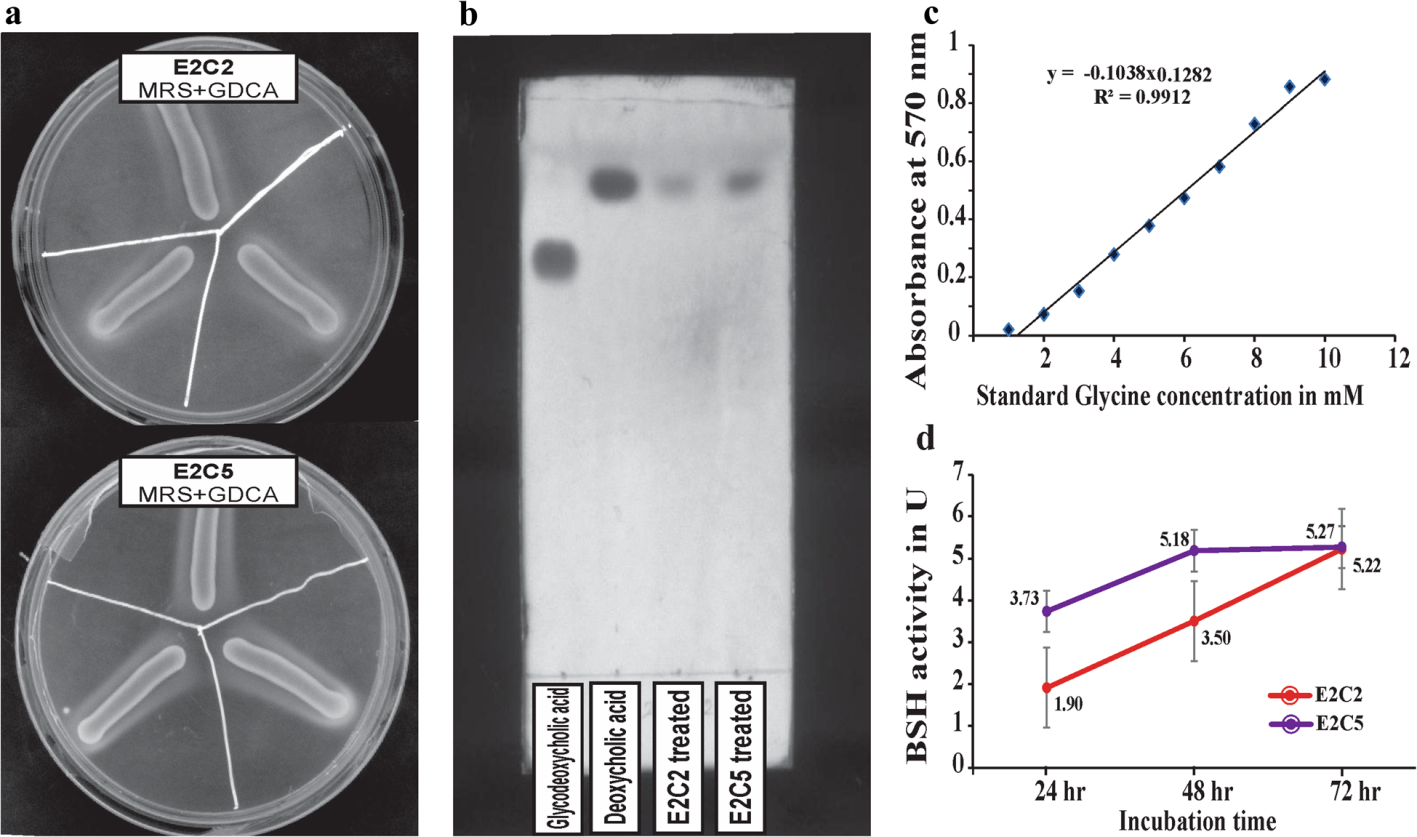
Bile salt hydrolase activity of *Lactobacillus plantarum* E2C2 and E2C5 isolates (**a**) Plate assay showing precipitation zones around the line of inoculation in triplicates (**b**) TLC plate assay showing deconjugation ability and (**c** & **d**) Ninhydrin assay indicating quantification of glycine removal by deconjugation ability

16S rRNA gene sequencing and *is*DDH were used for the identification for isolates. 16S rRNA gene sequences were used for phylogenetic analysis using neighbour-joining method, which reveals that the two isolates E2C2 and E2C5 isolates are the members of *Lactobacillaceae* family, including *Lactobacillus plantarum* WCFS1, a previously reported probiotic bacterium isolated from human saliva [23] and *Lactobacillus plantarum* strain 5*–*2 [24], earlier isolated and identified from fermented foods (Fig. 3). The *is*DDH analysis was performed against type strain *L. plantarum* ATCC 14197 ^T^ for ANI and GGDC [25, 26]. Both the isolates congruently showed 98.91% ANI and 93.60% GGDC score to the type strain, which are more than recommended thresholds (95% for ANI and 70% for GGDC) for the identification of the species, confirming both isolates as *L. plantarum*, belonging to the phylum *Firmicutes* and class *Bacilli*. Both the strains are deposited in National Collection of Industrial Microorganisms, Pune with accession no. NCIM 5603 (*L. plantarum* E2C2) and NCIM 5602 (*L. plantarum* E2C5). The isolates were also deposited in Microbial Culture Collection, Pune with accession no. MCC 3016 (*L. plantarum* E2C2) and MCC 3190 (*L. plantarum* E2C5).

## Genome sequencing information

### Genome project history

The isolates were selected for sequencing as part of an ongoing project investigating the association of gut microbiota with hyperoxaluric condition. Based on metabolic versatility and oxalate tolerance, strains E2C2 and E2C5, were selected and sequenced by Illumina MiSeq platform at Institute of Medical Microbiology, Germany. This Whole Genome Shotgun project has been deposited at DDBJ/EMBL/GenBank under the accession LSST00000000.1 and LTCD00000000.1 (Table 2). The version described in this paper is version LSST00000000.1 and LTCD00000000.1.

**Table 2:** Project information

MIGS ID	Property	*L. plantarum* E2C2	*L. plantarum* E2C25
MIGS 31	Finishing quality	High-quality draft	High-quality draft
MIGS-28	Libraries used	300 bp	300 bp
MIGS 29	Sequencing platforms	Illumina MiSeq	Illumina MiSeq
MIGS 31.2	Fold coverage	100 ×	100 ×
MIGS 30	Assemblers	DNASTAR assembler v. 11.2.1.25	DNASTAR assembler v. 11.2.1.25
MIGS 32	Gene calling method	RAST	RAST
	Locus Tag	AYO51	AZJ01
	Genbank ID	LSST00000000.1	LTCD00000000.1
	GenBank Date of Release	03/23/2016	03/25/2016
	GOLD ID	Gs0118511	Gs0120378
	BIOPROJECT	PRJNA311909	PRJNA313343
MIGS 13	Source Material Identifier	NCIM 5603, MCC 3016	NCIM 5602, MCC 3190
	Project relevance	Human stool bacteria	Human stool bacteria

### Growth conditions and genomic DNA preparation

The E2C2 and E2C5 bacterial strains of *L. plantarum* were cultured in MRS agar (MA; Difco) at 30 °C under the aerobic condition for 3 days of incubation. Genomic DNA of the bacterial strains were isolated using a Qiagen DNA extraction kit (Hilden, Germany) following manufacturer’s instructions. Extracted DNA quality was assessed by 1.0% agarose gel electrophoresis, concentration and purity (A_260_/A_280_) were measured using NanoDrop ND-1000 (NanoDrop technologies, Willingminton, USA). Extracted DNA samples of the strains were preserved at −20 °C until further processing.

### Genome sequencing and assembly

The bacterial genomes of *L. plantarum* E2C2 and *L. plantarum* E2C5 were sequenced by Illumina MiSeq platform using 2x300 paired-end libraries. Sequence quality of both the genomes was analyzed for quality control using FastQC software [27]. After analysis, raw sequences were trimmed and assembled using *de novo* assemblers SPAdes 3.5.0 [28] and DNA star assembler v. 11.2.1.25. More than 6 million good quality paired-end reads were obtained from both the strains, which accounted for an approximate 100x sequencing coverage. After assembly, it was found that the draft genomes of *L. plantarum* E2C2 and *L. plantarum* E2C5 contained 94 and 99 scaffolds respectively.

### Genome annotation

Assembled genomes of both the strains were annotated using RAST version 2.0 [29] and the NCBI Prokaryotic Genome Annotation Pipeline [30]. Protein-encoding genes, tRNA and rRNA genes of the genomes were predicted using Glimmer version 3.02 [31], tRNA_scan-SE [32], and RNAmmer [33], respectively. Protein coding genes were analyzed by COG database [34] on WebMGA [35] and Pfam domains were predicted using NCBI Batch CD-Search Tool [36]. Transmembrane helix and signal peptide prediction of the genome was identified by using Phobius [37]. The presence of CRISPR repeats was predicted using the CRISPRFinder tools [38] (Table 4).

## Genome properties

The draft genome sequence of *L. plantarum* strains E2C2 and E2C5 contained 3603,563 bp and 3615,168 bp, with GC content 43.99% and 43.97%, respectively. The reads of *L. plantarum* strains E2C2 and E2C5 were assembled into 94 and 99 contigs (*N*_50_, 235,913 bp, and 256,152 bp, respectively). The genome sequence of *L. plantarum* strain E2C2 included a total of 3504 genes and 3289 candidate CDS, giving a coding intensity of 94%. The genome was shown to encode at least 94 predicted RNAs, including 15 rRNAs and 75 tRNAs, and also 121 pseudogenes. Whereas, *L. plantarum* E2C5 genome which contained total 3523 genes and 3293 candidate CDS. *L. plantarum* E2C5 genome contained 95 predicted RNAs including 16 rRNAs and 75 tRNAs, and also 135 pseudogenes (Table 3). The draft genome size of the strains E2C2 and E2C5 was more than average of *L. plantarum* genome size that has been reported in public databases. It was found that most of the predicted genes (87.19% and 87.15% of strains E2C2 and E2C5, respectively) code for proteins which involved in major metabolic pathways were assigned to one of the 25 functional COG categories while the remaining genes were assigned as unknown functional proteins (Table 4).

**Table 3:** Genome statistics

Species Attribute	*L. plantarum* E2C2		*L. plantarum* E2C5	
	Value	% of Total	Value	% of Total
Genome size (bp)	3,603,563	100.00	3,615,168	100.00
DNA coding (bp)	2,684,877	74.5	2690, 385	74.4
DNA G + C (bp)	1,585,330	43.9	1589, 803	43.9
DNA scaffolds	94		99	
Total genes	3504	100	3523	100
Protein coding genes	3289	93.8	3293	93.4
RNA genes	94	2.6	95	2.6
Pseudo genes	121	3.4	135	3.8
Genes in internal clusters	NA		NA	
Genes with function prediction	2416	68.9	2426	68.9
Genes assigned to COGs	2868	81.8	2869	81.4
Genes with Pfam domains	2952	89.7	2969	90.1
Genes with signal peptides	278	7.9	275	7.8
Genes with transmembrane helices	755	21.5	755	21.4
CRISPR repeats	1	0.028	1	0.028

**Table 4:** Number of genes associated with general COG functional categories

Code	*Lactobacillus plantarum*					Description
	E2C2		E2C5		WCFS1	
	Value	% age	Value	% age	Value	
J	213	6.47	213	6.47	197	Translation, ribosomal structure and biogenesis
A	0	0	0	0	0	RNA processing and modification
K	313	9.51	310	9.41	259	Transcription
L	166	5.04	168	5.10	103	Replication, recombination and repair
B	0	0	0	0	0	Chromatin structure and dynamics
D	49	1.48	48	1.45	33	Cell cycle control, Cell division, chromosome partitioning
V	92	2.79	92	2.79	76	Defense mechanisms
T	106	3.22	104	3.15	86	Signal transduction mechanisms
M	186	5.65	184	5.58	158	Cell wall/membrane biogenesis
N	22	0.66	22	0.66	10	Cell motility
U	26	0.79	26	0.78	17	Intracellular trafficking and secretion
O	99	3.01	100	3.03	83	Posttranslational modification, protein turnover, chaperones
C	118	3.58	118	3.58	101	Energy production and conversion
G	286	8.69	286	8.68	265	Carbohydrate transport and metabolism
E	218	6.62	219	6.65	183	Amino acid transport and metabolism
F	103	3.13	104	3.15	89	Nucleotide transport and metabolism
H	116	3.52	116	3.52	86	Coenzyme transport and metabolism
I	97	2.94	96	2.91	80	Lipid transport and metabolism
P	121	3.67	121	3.67	105	Inorganic ion transport and metabolism
Q	19	0.57	20	0.60	23	Secondary metabolites biosynthesis, transport and catabolism
R	189	5.74	189	5.74	174	General function prediction only
S	235	7.14	236	7.16	201	Function unknown
X	94	2.85	97	2.94	55	Mobilome: prophages, transposons
-	421	12.80	424	12.87	-	Not in COGs

The total is based on the total number of protein coding genes in the genome

## Insights from the genome sequences

Genome sequence analysis of *L. plantarum* strains E2C2 and E2C5 showed a presence of common subsystem structure, *i.e.*, carbohydrate and protein metabolisms, iron acquisition and metabolism, chemotaxis, stress response, secondary metabolism, nitrogen metabolism, dormancy and sporulation. Genome analysis of both the strains showed that more than 800 genes are present for carbohydrate metabolism indicating a diverse carbohydrate utilization pattern or abilities that include C1- metabolism, organic acids, mono-, di- and polysaccharides metabolisms. *Lactobacillus* is well known for its capability to grow in protein-rich environments and contains protein degradation enzymes/machinery, therefore it is well adapted to these conditions. It was observed that both the strains have more than 50 protein degrading enzymes/transport systems that include metallo-carboxypeptidases, dipeptidase, proteasome and many ATP-dependent uptake systems. A large number of stress response systems that include oxidative stress, heat shock and cold shock are present in both the strains. Stress response genes, namely sodA, sodB, HPI, HPII and CCP for reactive oxygen species; PRP, Rex, OxyR, Fnr, ZUR and FUR for oxidative stress; HrcA, GrpE and fam for heat shock response were identified. In *L. plantarum* strains E2C2 and E2C5, genes for alpha-glucosidase, choloylglycine hydrolase, alpha-L-rhamnosidase essential for antidiabetic, hydrolysis of bile salt in the small intestine, adaptation to changing nutritional resources are noted. Therefore, the analysis suggests that both the *L. plantarum* strains (E2C2 and E2C5) can be used in multi-therapeutic aspects. The presence of biotin and other cofactors, vitamins, prosthetic groups and pigment synthesis genes are observed in the genome of both the strains, suggesting their ability to produce bioactive compounds. Considerable variation was not observed in the remaining subsystems that indicates biochemical homogeneity and similar capabilities of the strains in substrate utilization and processing. In addition, both *L. plantarum* E2C2 and *L. plantarum* E2C5 contain sulfur cycling, cobalt, zinc, and cadmium resistance genes.

### Extended insights

Comparison of the strains E2C2 and E2C5 genome showed 99.99% shared CDS and only 112 SNPs among the core genome, thus overall demonstrating the high similarity of the two genomes (Tables 3 and 4). The high similarity of the two isolates, despite the different source of isolation, is an indication of their selective adaptation to the gut environment. But based on COG data analysis it was found that these two strains E2C2 and E2C5 were differed from each other with respect to number of protein coding genes namely signal transduction mechanisms, cell wall/membrane biogenesis, Mobilome: prophages, transposons, etc. Oxalate tolerance ability of the two isolates is an important feature to note. In the hyperoxaluric condition, human gut often acts as a primary excretory organ of oxalate [39] and higher oxalate concentration in the gut has been linked with dysbiosis [20]. In the light of oxalate tolerance ability of the E2C2 and E2C5 isolates, their use as probiotics for hyperoxaluric patients is anticipated. In addition, genomes of strains E2C2 and E2C5 were compared with the reference strain, *Lactobacillus plantarum* WCFS1 [17]. The comparison revealed that the three genomes comprised 2639 genes in common at 80% coverage and 90% sequence identity [40]. E2C2 and E2C5 both contained an additional 345 genes while WCFS1 strain contained additional 265 genes. Further, about 344 genes were exclusively found in strains E2C2 and E2C5 as compared to strain WCFS1. When COG categories compared, a significant difference was observed for the functional annotation of the genes. COGs functional categories could be assigned to 2868 and 2869 genes for E2C2 and E2C5 respectively, while in case of WCFS1 only 2384 genes could be categorised by COGs (Table 4).

## Conclusions

Considering the high genetic versatility of *Lactobacillus plantarum* [14], it is important to sequence as many strains as possible to account for the genetic variability and their association with specific probiotic features such as oxalate tolerance. In this study, we provide the in-depth genome analysis of two oxalic acid and bile acid tolerant isolates- *L. plantarum* E2C2 and *L. plantarum* E2C5 obtained from healthy human stool samples. Genomic as well as phenotypic analysis reveals that both the isolates are coherent belonging to a single genetic lineage. The two strains described here can be an intriguing target to be explored further for their probiotics potentials in managing the specific metabolic disorders such as hyperoxaluria.

## Abbreviations

ANI: Average nucleotide identity
CDS: Coding DNA sequences or protein coding genes
GGDC: Genome-to-genome distance calculator
GRAS: Generally regarded as safe
*is*DDH: *In silico* DNA-DNA hybridisation
MRS medium: De Man, Rogosa and Sharpe medium
